# Emotion sensitivity and self‐reported symptoms of generalized anxiety disorder across the lifespan: A population‐based sample approach

**DOI:** 10.1002/brb3.1282

**Published:** 2019-04-16

**Authors:** Lauren A. Rutter, Luke Scheuer, Ipsit V. Vahia, Brent P. Forester, Jordan W. Smoller, Laura Germine

**Affiliations:** ^1^ Institute for Technology in Psychiatry McLean Hospital Belmont Massachusetts; ^2^ Department of Psychiatry Harvard Medical School Belmont Massachusetts; ^3^ Division of Depression and Anxiety Disorders McLean Hospital Belmont Massachusetts; ^4^ Division of Geriatric Psychiatry McLean Hospital Belmont Massachusetts; ^5^ Psychiatric and Neurodevelopmental Genetics Unit, Center for Genomic Medicine Massachusetts General Hospital Boston Massachusetts; ^6^ Stanley Center for Psychiatric Research, Broad Institute Cambridge Massachusetts

**Keywords:** emotion perception, generalized anxiety disorder, lifespan

## Abstract

**Background:**

Individuals with generalized anxiety disorder (GAD) symptoms show deficits in emotion processing, but results of prior studies have been conflicting, and little is known about developmental trajectories of emotion processing over time. We examined the association between GAD symptoms and sensitivity to recognizing emotional facial expressions (emotion sensitivity: ES) for three emotions (happiness, anger, fear) in a large, diverse, population‐based sample. We hypothesized that higher anxiety scores would be associated with poorer performance, and expected that ES performance and anxiety scores would decline across the lifespan.

**Method:**

Participants were 7,176 responders to a web‐based ES study (age range = 10–96 years old).

**Results:**

Higher GAD‐7 scores were associated with poorer ES performance for all emotion categories (happiness, anger, fear). The relationship between GAD‐7 and ES scores remained significant after controlling for the effects of age and sex, and there was no significant interaction, indicating that the relationship does not change across age. Age significantly predicted ES and GAD‐7 scores across emotions, with older ages showing lower ES scores and lower anxiety.

**Conclusions:**

In the largest study of its kind, GAD symptoms were associated with impaired ES performance across three emotion types. Future research should explore the connection between anxiety symptoms, cognitive processing, and social processing to better characterize the mechanisms of how GAD is linked with both social and non‐social information processing. Future work may also look at if ES is related over time to changes in anxiety, making it a promising target for intervention.

## 1 INTRODUCTION

For the last 40 years, researchers have investigated the connection between emotional disorders like anxiety and mood disorders and cognitive, perceptual, and emotional biases (e.g. Beck, 1976; Cisler, Olatunji, Feldner, & Forsyth, 2010; Eysenck, 2013; Leppänen, 2006; MacLeod, Mathews, & Tata, 1986; Mathews & Mackintosh, 1998; Mogg, Millar, & Bradley, 2000). Accurate identification of others’ emotions is critical for the initial development and progression of interpersonal relationships. Emotion perception has several components including (a) appraisal and identification of the emotional significance of a stimulus, (b) production of an affective state in response, and (c) regulation of the affective state and emotional behavior (see Phillips, Drevets, Rauch, & Lane, 2003 for a critical review). Here, we focus on one aspect of appraisal and identification of emotion: facial emotion sensitivity (ES), or, the ability to detect and discriminate intensities of emotional facial expressions. Previous studies have linked ES with higher interpersonal functioning and better quality of life (Davis, 1983; Mueser et al., 1996). Because emotion perception is reliant on complex and widespread neuropsychological processes, it can be disrupted both by psychiatric disorders such as anxiety, and by the natural aging process (Adolphs, 2002; Ruffman, Henry, Livingstone, & Phillips, 2008; Vrijen et al., 2016; Zhuang et al., 2018).

Previous research findings about the effects of generalized anxiety disorder (GAD) on emotion perception been varied and even contradictory. While some studies have linked GAD with lower emotion identification ability (Attwood et al., 2017) and selective attention to threat relative to controls, other studies have shown people with generalized anxiety symptoms have a lower threshold for emotion identification, that is, they perceive target emotions more quickly than non‐anxious groups (Bradley, Mogg, White, Groom, & de Bono, 1999; Bui et al., 2017; Plana, Lavoie, Battaglia, & Achim, 2014). High trait anxiety has been linked to better facial recognition of fear specifically (Surcinelli, Codispoti, Montebarocci, Rossi, & Baldaro, 2006). A meta‐analysis of impaired attribution of emotion to facial expressions showed that adults with anxiety disorders show moderate impairment in facial emotion recognition (*d* = −0.58, *p* < 0.001) (Demenescu, Kortekaas, den Boer, & Aleman, 2010). However, this same meta‐analysis showed that children with anxiety disorders do not have an overall deficit in recognizing emotions (Demenescu et al., 2010). Thus, the relationship between ES and anxiety may differ across the lifespan.

As with anxiety symptoms, aging also impacts a person's ability to correctly define a given emotion. Based on a meta‐analysis conducted by Ruffman et al., (2008), older adults are slightly better than young adults at recognizing expressions of disgust, but worse at detecting anger, sadness, fear, and happiness. Emotional identification ability also appears to increase during the transition to adulthood, with people below the age of 15 showing a lower perception ability than those between the ages of 15 and 30 (Olderbak, Wilhelm, Hildebrandt, & Quoidbach, 2018).

There are several limitations to the existing literature including methodological problems (e.g. Macmillan & Creelman, 2004), conflicting results (see Ko, 2018 for a review), and concerns with achieving adequate power and replicability (e.g. Asendorpf et al., 2013). We address these problems by using a large, diverse sample assessed via the web‐based laboratory, TestMyBrain.org (see Germine et al., 2012). Web‐based testing methods allow for the feasible recruitment of large samples across a broad age range. Previous comparisons of emotion perception tests administered on the web versus in the lab have shown that data are comparable in quality (Germine et al., 2012; Hartshorne & Germine, 2015; Meyerson & Tryon, 2003).

Thus, we aimed to resolve this mixed literature by (a) studying a much larger and more diverse sample than previously studied; (b) using a method that targets ES, a specific aspect of emotion perception; and (c) accounting for the potentially confounding effects of age on ES. Importantly, we also used a bias‐free method to obtain sensitivity scores, using two‐alternative forced choice intensity judgments for individual emotions. This allowed us to experimentally isolate ES for each emotion (happiness, anger, fear), free of potentially confounding differences in response bias that might also vary with age and anxiety symptoms. We hypothesized that our experimental design and well‐powered sample might uncover previously unobservable associations between anxiety and ES. We had two primary hypotheses: (a) higher anxiety will predict reduced sensitivity to all emotions (happiness, fear, anger) and (b) anxiety and ES will both decrease across the lifespan. We also tested the interactions between age, anxiety, and ES, expecting that the relationship between ES and anxiety would not be age dependent.

## 2 METHOD

### 2.1 Participants

Participants were 7,176 visitors to TestMyBrain.org who completed three ES tasks and the GAD‐7 (Spitzer, Kroenke, Williams, & Lowe, 2006) between January 2018 and June 2018. TestMyBrain.org is an online testing tool where participants take part in research experiments to contribute to science and learn more about themselves through immediate and personalized return of research results. Participants provided electronic consent to be a part of the study. Study and informed consent procedures were reviewed by the Harvard Committee on the Use of Human Subjects. After completing the task, participants were given feedback about their performance relative to other individuals who had completed the same measures.

Participants’ ages ranged from 10 to 96 years old, and the average age was 30.13 (standard deviation [*SD*] = 14.83). Females comprised the largest proportion of our sample (57.44%; male = 41.03%; missing = 1.0%; gender queer = 0.5%). The majority of participants self‐identified as Caucasian (64.3%) from English speaking countries. Highest completed education levels were: high school (24.20%), some college (22.77%), college (19.71%), and graduate school (17.25%).

### 2.2 Measures

#### 2.2.1 Belmont Emotion Sensitivity Test

We used the Belmont Emotion Sensitivity Test (BEST; see Rutter et al., 2019) to examine ES across the lifespan. The BEST was designed to eliminate response bias‐related confounds and allow us to match different emotion categories in terms of difficulty and reliability. Facial stimuli were drawn from the Karolinska Directed Emotional Faces database (Lundqvist, Flykt, & Öhman, 1998). Faces were morphed between any two of angry, happy, and fear, across a set of over two dozen identities, creating three morph continua per identity. Sensitivity to anger, happiness, and fear were assessed separately across three subtests. For each subtest, participants were shown 56 pairs of faces, one pair at a time, with the two faces in a pair presented on screen at the same time, for 1,000 ms. Participants were asked to indicate, “Which face is more happy?”, “Which face is more angry?”, or “Which face is more fearful?” to evaluate happiness ES, anger ES, and fear ES, respectively. Trials were ordered so that difficulty increased across three blocks (easy = 8, medium = 20, and hard = 28) for each subtest. For anger, there was a 70% difference along each morph continuum for easy faces, a 40% difference for medium difficulty faces, and a 20% difference for hard faces. For fear, there was an 80% difference for easy faces, a 50% difference for medium difficulty faces, and a 30% difference for hard faces. For happiness, there was a 70% difference for easy faces, a 30% difference for medium difficulty faces, and a 10% difference for hard faces. For example, for an easy anger trial with a 70% difference, one face might contain 90% of the anger face and 10% of the happy face (or 10% fear), while the other face might include 20% anger and 80% happiness (or 80% fear).

Participants were excluded from analyses if they had <50% accuracy (chance performance) or had mean reaction times (RTs) <200 ms (suggesting non‐compliance with the task). After binning ages by year, we excluded ages that had fewer than 10 participants, which restricted our age range from 11 to 69. Thus, our final sample size consisted of 7,066 participants who completed all three tests and the GAD‐7, and were in an age bin with at least 10 participants. For all emotion categories (i.e. happiness, fear, anger), lower ES scores are indicative of poorer performance, or lower sensitivity in recognizing a particular emotion. Compared to other tests of emotion recognition or ES, the BEST eliminates ceiling effects for happiness, and demonstrates high reliability (Rutter et al., 2019).

#### 2.2.2 Generalized Anxiety Disorder Questionnaire (GAD‐7; Spitzer et al., 2006)

The GAD‐7 is a 7‐item self‐report scale developed to assess the defining symptoms of GAD. Items are rated on a 4‐point Likert‐type scale (0 = “not at all” to 3 = “nearly every day”). Scores range from 0 to 21 with higher scores representing more severe generalized anxiety symptoms. Research has suggested that the GAD‐7 is a valid screening tool for GAD in a primary care setting and in the general population (Lowe et al., 2008; Spitzer et al., 2006). Internal consistency of GAD‐7 scores, measured by Cronbach's alpha, in our sample was 0.89.

### 2.3 Data analysis

Data were analyzed in R. Effect sizes are reported with 95% CIs. First, we examined bivariate correlations to confirm that ES and GAD‐7 both varied with age and sex. Next, we tested our specific hypotheses using linear regression. Last, we conducted follow‐up regression analysis to examine the interactions between variables. For each major analysis, we corrected for multiple comparisons (three emotion categories) using a conservative Bonferroni adjustment (*p* < 0.017 uncorrected). For clarity, all analyses are reported with uncorrected *p*‐values. Results were only considered statistically significant, however, if they survived correction for multiple comparisons (*p *< 0.05 corrected). Results that survived correction are indicated, and interpretation of the findings are based on this.

## 3 RESULTS

### 3.1 Descriptive data

*Anger:* The average ES accuracy for anger was 0.85 (*SD* = 0.09). Average mean RT, median RT, and *SD* RT in milliseconds were 1,226.35, 1,178.56, and 318.53, respectively. *Happiness:* The average ES accuracy for happiness was 0.84 (*SD* = 0.07). Average mean RT, median RT, and *SD* RT were 1,216.78, 1,169.57, and 347.40, respectively. *Fear:* The average ES accuracy for fear was 0.80 (*SD* = 0.11). Average mean RT, median RT, and *SD* RT were 1,468.56, 1,405.43, and 373.28, respectively. *GAD‐7:* The average GAD‐7 score in our sample was 7.89 (*SD* = 5.40, range = 0–21), which is consistent with mild GAD symptoms.

Based on Welch's *t* tests, accuracy scores significantly differed by category with anger accuracy significantly better than fear (*t* = 30.76, *p* < 0.001, *d* = 0.50) and happiness (*t* = 7.89, *p* < 0.001, *d* = 0.12), and happiness significantly better than fear (*t* = 26.24, *p* < 0.001, *d* = 0.43). Additionally, response speeds differed by emotion category, with fear slower than anger (*t* = 53.94, *p* < 0.001, *d* = 0.91) and happiness (*t* = 55.17, *p* < 0.001, *d* = 0.93), and a small, but nominally significant difference between anger and happiness (*t* = 2.25, *p < 0.0*5, *d* = 0.04), with happiness faster than anger. However, only the relationship between fear and the other two emotions survived correction.

### 3.2 Main effects of anxiety, age, and gender on ES

**Figure 1:**
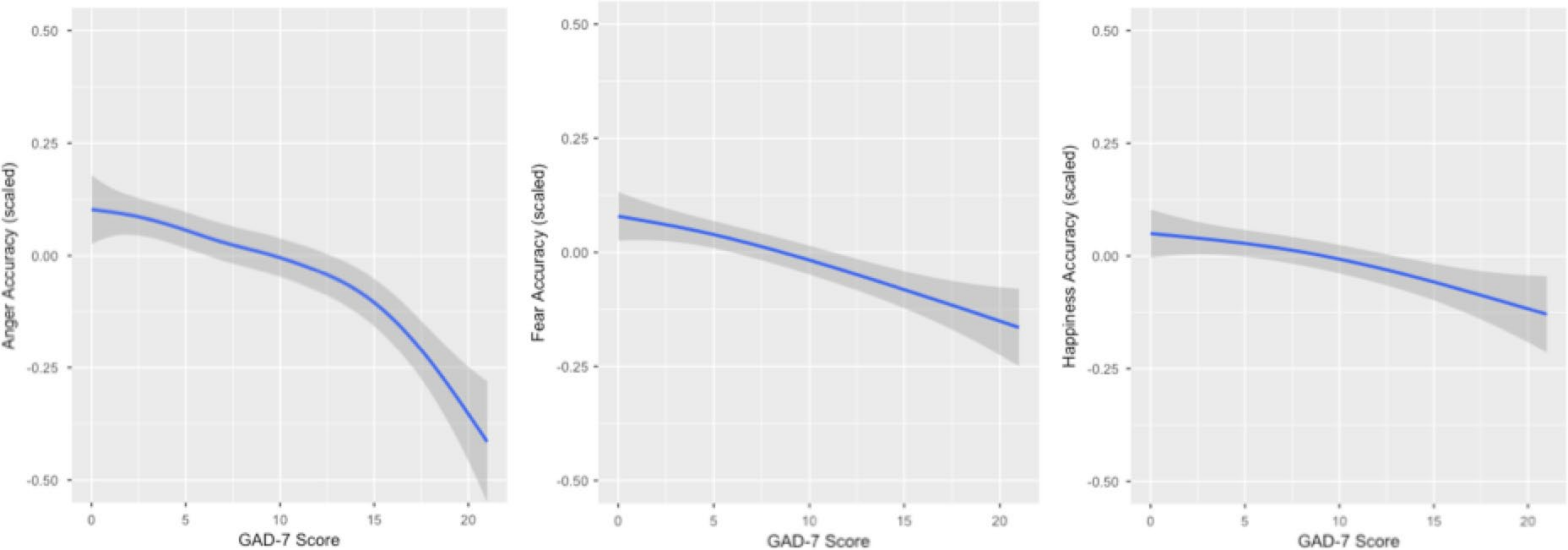
The effect of GAD‐7 score on emotion sensitivity *Note.* For visualization purposes, data are plotted with loess curve fitting using ggplot, a method for smoothing using local polynomial regression, using a single data point (mean) for each GAD‐7 score. Emotion sensitivity scores are scaled and plotted with 0.5 standard deviations. GAD‐7 scores significantly predicted emotion sensitivity scores across categories (anger = *R* ^2^ = 0.01, *F* (1, 7,064) = 65.95, *p* < 0.001; happiness = *R* ^2^ = 0.002, *F* (1, 7,064) = 14.12, *p* < 0.001; fear = *R* ^2^= 0.005, *F* (1, 7,064) = 35.39, *p* < 0.001). GAD, generalized anxiety disorder

The relationship between GAD‐7 score and ES accuracy was significant for each emotion category based on a series of univariate linear regressions of GAD‐7 score on ES category (anger = *R* ^2^ = 0.01, *F* (1, 7,064) = 65.95, *p* < 0.001; happiness = *R* ^2^ = 0.002, *F* (1, 7,064) = 14.12, *p* < 0.001; fear = *R* ^2^= 0.005, *F* (1, 7,064) = 35.39, *p* < 0.001). Higher GAD scores were associated with lower accuracy across categories, consistent with hypothesis 1 (Figure 1). Additionally, using linear regression, the relationship between GAD‐7 score and average speed of performance was significant across most emotion categories (anger = *R* ^2^ = 0.003, *F* (1, 7,064) = 21.90, *p* < 0.001; happiness = *R* ^2^ = 0.004, *F* (1, 7,064) = 26.82, *p* < 0.001, with the exception of fear (*p* = 0.18)). Higher anxiety scores on the GAD‐7 were associated with significantly slower ES performance for anger and happiness. While effect sizes were small, this demonstrates that GAD‐7 related differences in accuracy were not accounted for by speed accuracy trade‐offs. Controlling for sex and age, the relationship between GAD‐7 scores and ES remained significant across emotion categories (anger β = −1.924e‐03, *p <* 0.001, fear β = −2.336e‐03, *p <* 0.001, happiness β = −8.038e‐04, *p* < 0.001).

**Figure 2:**
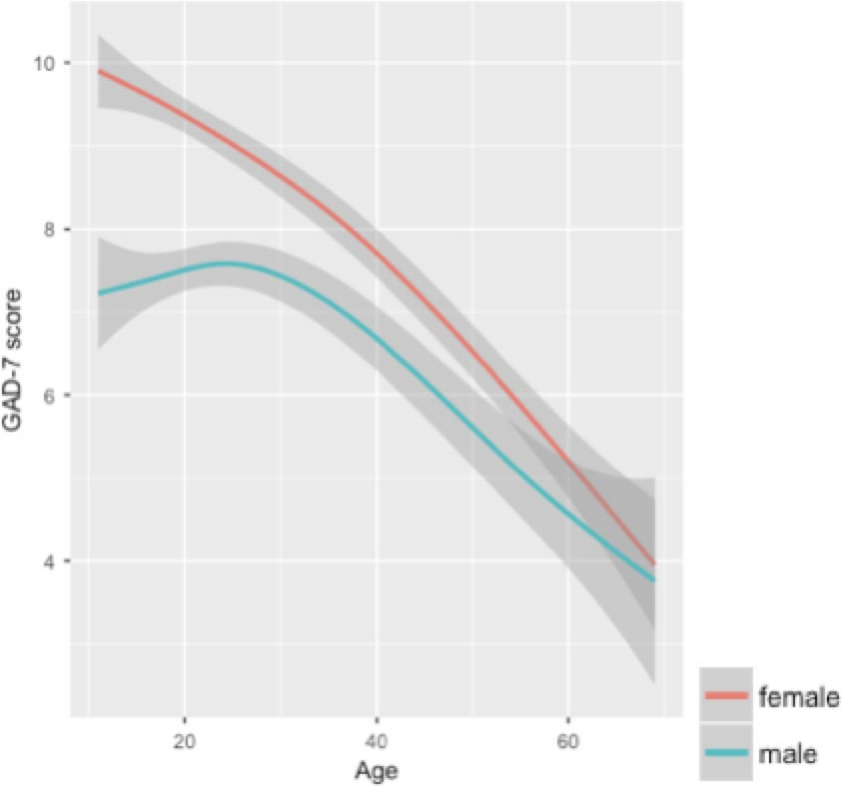
The effect of age and gender on GAD‐7 Score *Note.* For visualization purposes, data are plotted with loess curve fitting using ggplot, a method for smoothing using local polynomial regression, using a single data point (mean) for each age time point. GAD‐7 scores showed a significant decline across the lifespan for both genders (*R* ^2^ = 0.05, *F* (1, 7,062) = 116.00, *p* < 0.001). Males and females showed significantly different GAD‐7 scores, with women reporting higher levels of anxiety (*F* (1, 6,957) = 108.30, *p* < 0.001). GAD, generalized anxiety disorder

We also looked at the linear and nonlinear (quadratic and cubic) effects of age on ES. The relationship between age and ES was significant for fear (*R* ^2^ = 0.01, *F* (3, 7,062) = 21.61, *p* < 0.001), anger (*R* ^2^ = 0.02, *F* (3, 7,062) = 59.33, *p* < 0.001), and happiness (*R* ^2^ = 0.01, *F* (3, 7,062) = 12.79, *p* < 0.001). Though effects sizes were modest, these results suggest that older adults show decreased accuracy in recognizing fear, anger, and happiness, replicating results from Rutter et al. (2019) and consistent with Isaacowitz et al. (2007). Additionally, the relationship between age and GAD‐7 was significant (*R* ^2^ = 0.05, *F* (1, 7,062) = 116.00, *p* < 0.001), indicating that older participants had lower GAD symptoms (see Figure 2).

We conducted ANOVA to compare ES performance by sex. There were no significant sex differences for happiness or anger in either ES accuracy or speed. However, for fear sensitivity, females were more accurate than males (*F* (1, 6,957) = 46.60, *p* < 0.001) and also faster, based on their mean RT (*F* (1, 6,957) = 15.35, *p* < 0.05). Females also had significantly higher GAD‐7 scores compared to males (*F* (1, 6,957) = 108.30, *p* < 0.001) (Figure 2).

### 3.3 ES and GAD‐7 × age: interaction effects

The interaction between age and GAD‐7 score was not significant in predicting ES accuracy score for fear (*p* = 0.85) or happiness (*p =* 0.93); a small but nominally significant interaction effect was observed for anger (β = 4.886e‐07, *p <* 0.05), but did not survive the correction for multiple comparisons. This indicates that the relationship between GAD‐7 and ES did not significantly differ with age.

## 4 DISCUSSION

The purpose of this study was to examine the effect of age, sex, and GAD symptoms on ES in a large, diverse sample. ES provides an objective measure of social cognition, which may play a role in social functioning. While the effect of age on emotional facial processing has been repeatedly examined (see Ruffman et al., 2008 for a meta‐analysis), this is the first study to examine impact of anxiety symptoms across the lifespan on ES. ES was measured using an approach that did not depend on emotion categorization and allowed us to look at sensitivity to fear, happiness, and anger separately, with emotion judgments unconfounded by differences in response bias. This allowed us to address conflicting findings of prior literature, through improved methodology.

Results showed that there was a significant relationship between GAD‐7 scores and ES across emotions such that higher anxiety was associated with less accurate performance. This relationship was not dependent on age, suggesting such an effect is comparable across the lifespan. Age was generally associated with decreased accuracy in ES across the lifespan as well as decreased anxiety symptoms. Our findings are in line with other studies showing that people with high trait‐anxiety show slower responding to angry faces (Fox, Calder, Mathews, & Yiend, 2007), and in slight contrast to more recent reports that individuals with GAD show enhanced detection of anger (Ashwin et al., 2012). Overall, our findings replicate some prior findings on ES, while contributing novel insights about the effect of anxiety on ES at various stages of life.

Results also showed that males and females differed significantly in their ES performance for fear, but not for anger or happiness. Females showed significantly heightened fear sensitivity than males, although the effect size was small. These findings provide additional support to the existing literature on sex differences in emotion perception, with females generally showing an advantage over males according to a meta‐analytic review (McClure, 2000). Our finding of heightened fear sensitivity in women is consistent with a recent study showing larger responses in women to subthreshold fearful faces based on an event‐related potential design (Lee, Kim, Shim, & Lee, 2017). Gender differences in neural response, reflected in early processing stages for emotional faces, could also impact the outcome observed in this sample. However, a recent study examining sex differences in face recognition showed no significant differences between males and females in the magnitude of neural responses in any face‐processing region (Scherf, Elbich, & Motta‐Mena, 2017). Moreover, another new study demonstrated female advantage in reading facial expressions that was unaffected by expression intensity level or emotion category, indicating a general but not specific advantage (Wingenbach, Ashwin, & Brosnan, 2018). Of note, all three of these studies were conducted with relatively small samples sizes, limiting interpretation and generalizability. Our results suggest that a specific effect may exist, but such an effect is relatively small and future studies aiming to identify neural correlates with emotion processing related to gender may require larger samples.

One limitation of the study was our method of sampling. Our anxiety scores were drawn from the GAD‐7, a widely used measure of GAD symptoms, but self‐report measures should be interpreted with caution given the possibility of reporting bias and self‐selection effects. We did not conduct a clinician‐administered anxiety assessment. It is possible that participants with particularly high or low anxiety were drawn to doing this study. Selection of a truly random sample is difficult to achieve, and is a ubiquitous problem in the literature. We used an entirely web‐based approach where the impact of self‐selection biases is not understood as well. Such approaches are being used more commonly to recruit larger and more diverse samples (Fortenbaugh et al., 2015; Halberda, Ly, Wilmer, Naiman, & Germine, 2012; Hartshorne & Germine, 2015; Soto, John, Gosling, & Potter, 2011); however, more work is needed to understand how to best benefit from web‐based sampling while accounting for biases related to a nonrandom sample. Finally, high comorbidity between anxiety and mood disorders is another facet that may influence ES performance. A recent study on recognition of facial expressions in adults with comorbid depression and anxiety (*n* = 14) compared to nonanxious depression (*n* = 14) demonstrated that diminished sensitivity to happy and sad expressions was specific to anxious depression but not hypervigilance toward threatening facial expressions (Berg et al., 2016). In our sample, participants completed a GAD‐7 but no measures of depression, limiting our analyses. Additionally, we did not assess medical comorbidities. Future studies should include measures of depression in addition to anxiety to understand the impact of comorbid symptoms in large samples.

## 5 CONCLUSION

In the largest study of its kind, anxiety symptoms were shown to be associated with impaired ES across three emotion categories and across the lifespan. Our results provide insight into the importance of considering the effect of anxiety, age, and sex independently and in relation to each other when examining social processes. The effect of aging on ES should be explored in additional longitudinal studies with clinical and healthy populations. Future research should also examine the effect of an anxiety intervention on ES, and the effect of an ES intervention on anxiety. It is hypothesized that interventions targeted at enhancing ES could reduce anxiety, but this has not been tested. Given the intact relationship between aging, ES, and anxiety, it is possible that this intervention would be generalizable across the lifespan.

## CONFLICT OF INTERESTS

None declared.
